# Association between Urinary Sodium Excretion and Body Fat in School-Aged Children: Insights from the ARIA Study

**DOI:** 10.3390/nu16081197

**Published:** 2024-04-17

**Authors:** Ana Patrícia Soares, Mónica Rodrigues, Patrícia Padrão, Carla Gonçalves, André Moreira, Pedro Moreira

**Affiliations:** 1 Faculty of Nutrition and Food Sciences, University of Porto, 4150-180 Porto, Portugal; anapatriciarsoares@gmail.com (A.P.S.); patriciapadrao@fcna.up.pt (P.P.); andremoreira@med.up.pt (A.M.); 2 Epidemiology Research Unit, Institute of Public Health, University of Porto, 4050-600 Porto, Portugal; carolina101997@hotmail.com (M.R.); carlagoncalves@utad.pt (C.G.); 3 Laboratory for Integrative and Translational Research in Population Health, Institute of Public Health, University of Porto, 4050-600 Porto, Portugal; 4 CITAB—Centre for the Research and Technology of Agro-Environmental and Biological Sciences, University of Trás-os-Montes and Alto Douro, 5000-801 Vila Real, Portugal; 5 Basic and Clinical Immunology, Department of Pathology, Faculty of Medicine, University of Porto, 4200-319 Porto, Portugal; 6 Immuno-Allergology Department, São João Hospital Center, 4200-319 Porto, Portugal

**Keywords:** obesity, children, sodium, energy intake, body fat mass percentage, body mass index

## Abstract

Childhood obesity has been associated with increased sodium intake. Nonetheless, evidence linking sodium intake to Body Mass Index (BMI) and Body Fat Mass Percentage (%BF) remains limited, especially in the pediatric age group. Therefore, this study aims to investigate whether there is an association between 24 h urinary sodium excretion with BMI and %BF in a sample group of children from the ARIA study. This cross-sectional analysis included 303 children aged 7 to 12 from across 20 public schools in Porto, Portugal. Weight and %BF were assessed using the Tanita™ BC-418 Segmental Body Analyzer. Children’s Total Energy Intake (TEI) was estimated through a single 24 h Recall Questionnaire, and urinary sodium and potassium excretion was estimated by a 24 h urine collection. The association of %BF and BMI with 24 h sodium excretion was estimated by a binary logistic regression adjusted for sex, age, physical activity, total energy intake, parental education, and 24 h urinary excreted potassium. There was a significant positive association between higher levels of urinary sodium excretion and higher %BF values, even after adjusting for confounders. However, the same was not observed for BMI. Our findings suggest that higher sodium intake is associated with higher values of %BF among children, regardless of TEI and potassium intake.

## 1. Introduction

Childhood obesity is a complex public health issue affecting most developed countries and can be characterized by an excessive or abnormal accumulation of body fat [1,2]. This chronic disease is responsible for a series of complications that contribute to increased morbidity and premature mortality, with inadequate nutrition and physical inactivity being the main contributors to its cause [2].

Sodium intake has emerged as a critical aspect of childhood nutrition, and numerous studies have been conducted in different countries showing that children and adolescents tend to consume more sodium than recommended [3]. This trend is particularly concerning given the well-established association between high sodium intake and elevated blood pressure, a significant precursor to the development of cardiovascular diseases [4,5]. Furthermore, there has been a notable escalation in childhood obesity rates [6,7], concomitant with heightened sodium intake within this age group [8]. Based on the COSI Portugal 2022 study, despite a reversed trend in the prevalence of childhood overweightedness and obesity in Portugal from 2008 to 2019, the same was not observed in 2022. In fact, between 2019 and 2022, there was an increase of 2.2 and 1.6 percentage points in the prevalence of childhood overweightedness and obesity, respectively [9]. These trends represent significant epidemiological concerns warranting thorough investigation and targeted intervention strategies [6,7,8].

Evidence shows that it is highly recommended to estimate sodium intake from 24 h urinary excretion, as it is a more precise marker of sodium intake, which appears to be associated with adiposity [10] and an increased risk of obesity [11,12]. A cross-sectional study using the NHANES database demonstrated that the highest tertile of dietary sodium intake was positively associated with childhood obesity [13]. It has been demonstrated that an increase in salt intake by 1 g/day is associated with a 27 g/day increase in the consumption of sugary sodas in children and adolescents [14]. Therefore, this association between salt and obesity may be partly explained by the excessive consumption of energy-rich and salty processed foods [15]. However, growing evidence suggests that there may be a direct relationship between salt intake and obesity, independent of energy intake [16,17,18,19,20,21].

Body mass index (BMI) has been widely used in large-scale population research and public or clinical health screenings as a surrogate measure for fat mass content [22,23]. However, BMI may not be the most effective indicator for measuring adiposity [24], as evidenced in a meta-analysis where BMI showed high specificity but low sensitivity in detecting excess adiposity in children [25]. For this reason, the percentage of body fat mass (%BF) has been commonly used as a more reliable index of adiposity [22]. Moreover, for %BF, which is a better predictor of adverse health effects such as cardiovascular risk factors [26], obesity [27], and unfavorable lipid profiles [28], research remains scarce.

Thus, despite only a few studies demonstrating a direct association between sodium intake, and BMI and %BF, independent of energy intake [15,18,29,30], there have been none that used accurate measures of sodium and potassium intake in children. Taking this into consideration, this study aimed to examine the association between 24 h urinary sodium excretion, BMI, and %BF, independent of total energy intake, in a sample of school-aged children from the ARIA study.

## 2. Materials and Methods

### 2.1. Ethics

This study was approved by the Ethics Committee of the University Hospital São João (ARIA 248-13), and written consent was obtained from the legal guardian of each child. Furthermore, the research was conducted in accordance with the Helsinki Declaration.

### 2.2. Participants and Study Design

**Figure 1:**
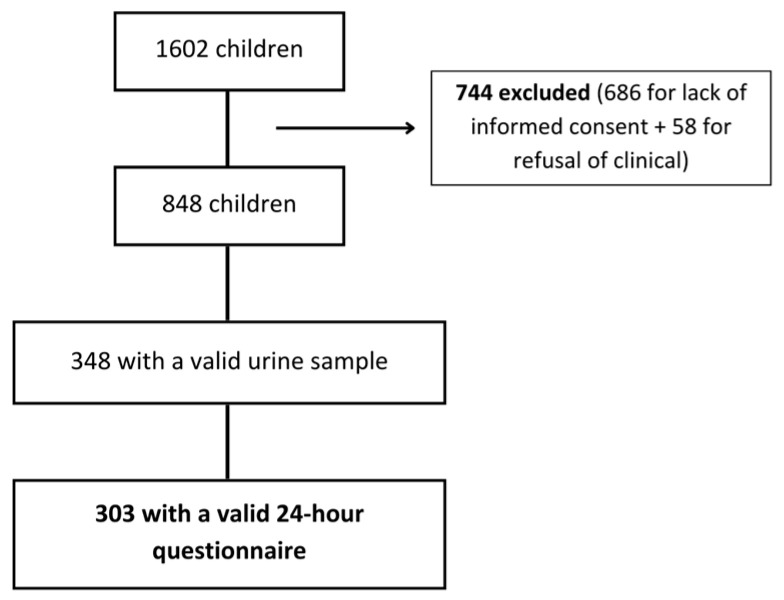
Summary of the participant inclusion process.

The population sample for this study was drawn from a cross-sectional survey carried out between January 2014 and March 2015. Specifically, 1602 children, aged between 7 and 12 years, enrolled in the 3rd and 4th grades across 20 public schools in Porto, Portugal, were extended invitations to participate [31]. Of the 1602 children, 686 (42.8%) did not provide signed consent, and 58 (3.8%) did not authorize clinical procedures. Among the remaining 858 children (53.6%), 348 (21.7%) had a valid urine sample, but only 303 (18.9%) were included in the analysis, as they were the only ones that had a valid 24 h food recall questionnaire (Figure 1).

### 2.3. Participant Assessment

#### 2.3.1. Total Energy Intake Assessment

The collection of dietary and nutritional information from the children was conducted by a trained interviewer using a single 24 h Food Recall Questionnaire. The interviewers assisted the children in recalling their dietary intake by prompting them to recall their previous day, starting from the time that they woke up and subsequently recalling their first meal of the day, continuing in chronological order, until the hour they went to bed. The data recorded per eating occasions included information about the time of consumption and the place. During the interview, participants were questioned in detail about all the foods and beverages they consumed, the quantities ingested, the methods of preparation, and commercial brands. In order to assist the children in estimating portion sizes of the ingested food from the previous day, a photographic atlas was used [32]. Food Processor^®^ software, manufactured by ESHA Research, Salem, OR, USA, SQL^®^, V3, which integrates detailed data on the nutritional composition of Portuguese foods, was used to estimate total energy intake (TEI). 

Subsequently, for the validation of the 24 h Recall Questionnaires, the Goldberg method was employed to detect and exclude “misreporters”, children with inaccurate reports in the questionnaire [33].

#### 2.3.2. 24 h Urine Collection

Regarding the 24 h urine collection, verbal and written instructions were provided to caregivers and parents to assist in collecting the 24 h urine sample from the children, along with a sterile urine collection container. On the first morning of collection, they were instructed to discard the initial sample and subsequently collect all samples, including the first sample of the next day, over a 24 h period. The collected samples were then sent for analysis to certified laboratories, where testing was conducted for 24 h creatinine (mg/day), 24 h urine volume (mL), 24 h potassium excretion (mEq/day), and 24 h sodium excretion (mEq/day). The values of sodium and potassium in the urine were converted to mg/day using the following formulas, respectively: 23 mg of Na^+^ = 1 mmol of Na^+^ or 1 mEq of Na^+^ [34], and 39.1 mg of K^+^ = 1 mmol of K^+^ or 1 mEq of K^+^ [35]. In order to assess the validity of the 24 h urine collections, creatinine coefficients were used, such as creatinine excretion in relation to weight, with creatinine coefficients above 0.1 mmol/kg/day classified as acceptable [36].

Urinary sodium levels were categorized into two distinct groups based on the median value observed within our sample. Specifically, individuals were categorized as either having lower urinary sodium levels (≤2507.00) or higher urinary sodium levels (>2507.00).

Additionally, for estimating salt intake, it was assumed that approximately 90% of consumed sodium is excreted in the urine [37].

#### 2.3.3. Anthropometric Measurements and Body Fat Mass Percentage

In terms of anthropometry, bioelectrical impedance analysis was applied using a digital scale (Tanita™ BC-418 Segmental Body Analyzer, Middlesex, UK) in order to obtain weight in kilograms and body fat percentage. %BF was assessed through the in-built Tanita equations. The children were barefoot and lightly dressed, and were instructed to stand still with their feet touching the metal plates. A portable stadiometer (SECA^®^ 214, Seca GmbH & Co., Hamburg, Germany) was used to measure their height in centimeters. BMI was calculated from weight and height data as the ratio of weight to height squared, in kilograms per square meter (kg/m^2^). Participants were then divided into two groups: non-overweight/obese (*p* < 85th) and overweight/obese (*p* ≥ 85th), based on specific BMI percentiles for sex and age from the US Centers for Disease Control and Prevention (CDC). This definition was used following an evaluation of the level of agreement among various BMI classifications, including those from the US CDC, the World Health Organization, the International Obesity Task Force, and Percentage of Body Fat. The analysis revealed that the BMI classification from the US CDC demonstrated the strongest agreement with all other classification systems [38]. Moreover, individuals were characterized using age- and sex-specific percentile curves for children’s body fat [39]. Thresholds were established at the 85th percentiles in order to delineate categories of under fat/normal fat (<85th) and overfat/obese (≥85th), respectively [39].

#### 2.3.4. Other Covariates

The evaluation of physical activity was conducted through a questionnaire distributed to each parent or guardian, and it included data on television/video viewing time, sleeping duration, and practice of sport activities besides the physical education classes at school (recorded in six frequency categories: “never”, “at least once per month”, “between once per month and once per week”, “2−3 times per week”, “4−6 times per week”, or “every day”). This last question was used to characterize children’s level of physical activity into three groups: <2 times/week, 2–3 times/week, or ≥4 times/week [40]. Parental education was also considered and categorized into three groups: ≤9 years, 10 to 12 years, and >12 years of education [41].

#### 2.3.5. Statistical Analyses

The statistical analysis was performed using SPSS^®^ software version 28.0. Initially, the normality of continuous study variables was assessed using skewness and kurtosis tests. Moreover, absolute and relative frequencies for all of the categorical variables were presented. For quantitative variables, the median and the 25–75 percentile range were chosen due to non-normal distribution. 

Subsequently, in order to investigate differences between children who were and were not overweight/obese, as well as with body fat percentage above or below the P85th, the Mann–Whitney test was used for quantitative non-normally distributed variables, and the Chi-square test was used for categorical variables.

Regarding the assessment of the association between BMI and %BF with urinary sodium excretion, a binary logistic regression model was employed (odds ratio (OR)), with a 95% confidence interval (CI). Finally, the null hypothesis was rejected when the significance level was less than 0.05 (*p* < 0.05).

## 3. Results

The characteristics of the participants included in this study are presented in Table 1, by the total number of participants (*n* = 303) and the following groups: non-overweight/non-obesity (*p* < 85th); overweight/obesity (*p* ≥ 85th); %BF considered to be “under fat/normal fat” (*p* < 85th); and body fat percentage considered to be “overfat/obese” (*p* ≥ 85th).

The median age of the children was 8.00 (8.00–9.00) years, and 53.5% (*n* = 162) were male. There were no significant differences between children who were not overweight/obese (*p* < 85th) and those who were overweight/obese (*p* ≥ 85th), except for 24 h urinary sodium excretion values [2392.00 (1886.00–3059.00) vs. 2725.50 (2087.25–3680.00)], and 24 h urinary potassium excretion levels [1642.20 (1290.30–2072.30) vs. 1915.90 (1485.80–2189.60)]. Regarding differences between groups based on %BF, children with higher %BF values exhibited significantly elevated 24 h urinary sodium excretion values compared to children with underfat/normal fat values [2300.00 (1863.00–3024.50) vs. 2760.00 (2236.75–3622.50)].

Regarding the relationship between BMI and excreted sodium, a significant positive association was observed in the crude model (OR = 2.04, 95% CI 1.18–3.50), which did not persist after adjusting for sex, age, total energy intake, physical activity, parental education, and urinary excreted potassium (aOR = 1.56, 95% CI 0.82–2.96). However, in the case of body fat percentage, %BF (*p* ≥ 85th), we can observe that children with increased urinary sodium excretion levels had higher odds of having higher %BF values in the crude model (OR = 2.62, 95% CI 1.56–4.38), as well as after adjusting for the previously referred to confounders, (aOR = 2.89, 95% CI 1.58–5.30). The results are presented in Table 2.

## 4. Discussion

This study presents novel findings showing that higher urinary sodium excretion levels were associated with an increased %BF in children, considering potential confounding factors such as energy intake and potassium urinary excretion, a crucial factor in sodium balance regulation. The relationship between obesity and sodium intake has been previously explained by increased liquid intake, particularly sugary drinks, and the excessive consumption of energy- and salt-rich processed products [12,15]. Therefore, energy intake could act as a confounding factor [42]. However, in our results, the association between sodium and %BF was independent of TEI. This is consistent with a study by Ma et al., which showed that a 1 g/day increase in salt intake was associated with a 0.73 kg (*p* = 0.001) increase in body fat mass in children, after adjusting for age, sex, ethnicity, and TEI [15]. 

The mechanisms for this positive association between sodium and %BF have not yet been fully elucidated, although some authors propose that excessive sodium intake may reflect unhealthy behaviors leading to adiposity [43], or even a direct contribution to an increase in body fat [44]. An experimental study in rats showed that the “high salt intake” group exhibited higher plasma leptin concentrations and an excessive accumulation of white adipose tissue compared to the lower salt intake group [45]. This could be explained by the improvement in insulin sensitivity of the adipocyte for glucose uptake, insulin-induced glucose metabolism, and the lipogenic capacity of white adipose tissue in rats [45,46]. Furthermore, a short-term increase in body weight due to sodium has also been demonstrated in various randomized clinical trials [47,48].

Similar results were observed in epidemiological studies [16,18,21,44]. A cross-sectional study involving adolescents was conducted in order to explore the association between dietary sodium intake, assessed using a seven day 24 h dietary recall method, and various adiposity measures. These measures included BMI, waist circumference, percent body fat, determined via dual-energy X-ray absorptiometry, subcutaneous abdominal adipose tissue and visceral adipose tissue, assessed using magnetic resonance imaging, and leptin levels, analyzed through fasting blood samples. Multiple linear regression analyses demonstrated independent associations between dietary sodium intake and adiposity measures, including body weight, BMI, waist circumference, percent body fat, fat mass, and subcutaneous abdominal adipose tissue, as well as leptin levels [16]. Another study, in children and adolescents, investigating the associations between urinary sodium excretion and dietary sodium intake with adiposity measures (BMI, waist circumference, and total body fat percent) demonstrated that higher urinary sodium excretion, as measured by spot urine analysis, exhibited significant associations with all adiposity measures, independent of energy intake, physical activity, sugar-sweetened beverage intake, and socio-demographic variables. As for dietary sodium intake, estimated by 24 h dietary recalls, increased Na consumption was consistently associated with increased odds of overweight and obesity, as indicated by BMI and waist circumference measures. Significant dose–response relationships were observed, indicating a linear trend of increasing adiposity with higher dietary sodium intake. However, no significant associations were found between dietary sodium intake and adiposity risk assessed by total body fat percentage [44]. Additionally, prospective cohort studies conducted in children, adolescents, and adults revealed that the initial values of 24 h urinary sodium excretion were associated with an increase in body fat percentage over time, independent of energy intake [18,21]. Therefore, these observations suggest that salt intake may exert a regulatory influence on body fat metabolism, potentially contributing directly to increased fat deposition. Elevated salt consumption appears to impact adipose tissue metabolism, as it has been demonstrated that high-salt diets may elevate plasma leptin concentrations and lead to an excessive accumulation of white adipose fat [15]. Furthermore, a pilot study showed that high sodium intake leads to cortisol production, and this increase in cortisol production appears to lead to obesity [49]. Such mechanisms align with the concept that dietary factors, beyond their caloric content, may intricately modulate metabolic pathways [15]. 

Despite our results not showing a significant association between BMI and urinary sodium excretion after adjusting for the mentioned confounders, a 2015 study found that BMI increased from the lowest to the highest tertile of salt intake (*p* < 0.001) in both children, after adjusting for age, sex, ethnicity, family income, physical activity, TEI, and inaccurate reports, as well as in adults, after additional adjustment for alcohol consumption, tobacco, and education level [15]. The absence of a statistically significant association between BMI and urinary sodium in our sample may potentially be attributed to the limitations of BMI in distinguishing between lean body mass and body fat mass. This suggests that BMI alone may not adequately capture the variability in body composition within our study sample. Consequently, an individual may exhibit a high BMI yet have low fat mass, or conversely, have a low BMI despite a higher proportion of fat mass. This discrepancy underscores the complexity of using BMI as a sole indicator of adiposity and highlights the importance of incorporating additional measures, such as body fat percentage or waist circumference, to more accurately assess adiposity status in children [50,51,52,53,54,55,56]. 

In the context of this cross-sectional investigation, our sample of school-aged children yielded a median sodium excretion of 2507 mg. Notably, this value is significantly above the recommendation for the age range of the study participants. The European Food Safety Authority (EFSA) recommends an intake of 1700 mg/day for ages 7–10 years and 2000 mg/day for that age onwards [57]. 

It is crucial to highlight the limitations inherent in our study design. Firstly, the reliance on a single 24 h urine collection may not fully capture an individual’s habitual sodium intake, as it can vary from day to day. Furthermore, the utilization of a sole 24 h recall questionnaire only permits assessment of short-term dietary intake, potentially overlooking seasonal fluctuations in food consumption. Therefore, it is imperative to acknowledge the need for a more comprehensive approach, such as employing multiple urine collections and various questionnaires in order to obtain a more reliable assessment of dietary intake [58]. Nonetheless, it is necessary to note that a considerable proportion of children/legal guardians did not provide informed consent, which may be due to the extensive and exhaustive nature of collecting a 24 h urine sample.

Dual-energy X-ray absorptiometry (DEXA) is considered to be one of the best methods for evaluating body composition [59]. In order to assess body fat, bioelectrical impedance analysis (BIA) was used, which, despite its limitations and not being considered a gold standard for body fat mass percentage assessment, is considered as a good alternative to DEXA [60,61,62,63]. Several studies that deemed BIA an inappropriate measure for evaluating body composition often had limitations in terms of age range and/or sample size [64,65,66]. Additionally, other authors found that BIA exhibited a high correlation and small differences compared to DEXA in assessing body fat mass, along with advantages related to its simplicity, portability, non-invasiveness, and speed [63,64,67,68,69,70]. Nonetheless, in the future, it would also be interesting to study other indicators of obesity such as waist circumference and waist-to-height ratio, as there is evidence suggesting that these measures demonstrate a good correlation with visceral abdominal fat [71] and metabolic risk factors in children [72,73]. 

It is also important to consider memory errors associated with the use of this type of questionnaire for dietary assessment, as participants may experience difficulties accurately recalling their dietary intake from the previous day. Furthermore, it is necessary to recognize possible limitations of knowledge regarding specific brands and to accurately estimate portions sized in this age group [74]. Additionally, it is essential to acknowledge that the utilization of a cross-sectional design in our study restricts our ability to establish a causal relationship between obesity and 24 h sodium excretion. This design limitation underscores the importance of interpreting our findings within the context of association rather than causation. While our study provides valuable insights into the relationship between these variables, it is imperative that the inherent limitations of cross-sectional research in delineating temporal sequences or determining causality are recognized [58]. While this study, as well as others, suggests that high salt intake is likely a contributing factor to obesity, we cannot also exclude the possibility that adiposity may predispose individuals to higher salt consumption independently of TEI [15]. 

However, it is important to underscore several strengths of our study methodology. Among these strengths is the utilization of 24 h urine collection, widely regarded as the gold standard method for assessing sodium and potassium intake. This robust methodology offers several advantages, including the ability to capture comprehensive data on individuals’ actual sodium and potassium excretion over a complete day, thereby minimizing potential biases associated with self-reported dietary assessments [37]. Furthermore, it is important to note the implementation of the creatinine coefficient as a measure to ensure the validity of the urine samples. This approach helps mitigate potential inaccuracies and collection errors that may arise with the previously mentioned method. By employing the creatinine coefficient, our study aimed to enhance the reliability of urine sample analysis, thereby bolstering the credibility of findings related to sodium excretion among participants [36]. It is important to emphasize the meticulous approach employed during the data collection process for dietary assessment. Trained interviewers were specifically tasked with conducting these assessments, ensuring that information was gathered in a systematic and unbiased manner. This rigorous methodology aimed to minimize potential sources of bias or error in dietary data collection, thereby enhancing the reliability and accuracy of the dietary information obtained from the children [75]. Additionally, it is important to highlight the advantage of the absence of potential modification of food intake associated with the use of a quick and retrospective information collection method. By employing such a methodology, participants are less likely to alter their eating behaviors or food choices in response to the data collection process. This minimizes the risk of bias introduced by participants’ awareness of being monitored or observed, thereby facilitating a more accurate characterization of children’s eating habits [75,76]. Furthermore, the dietary assessment process encompassed a comprehensive evaluation of various factors, as a wide range of specific product brands, portion sizes, and components of mixed dishes, which allowed us to capture a detailed representation of participants’ dietary food intake [75]. Finally, it is essential to highlight three additional strengths inherent in our research methodology. Firstly, the validation of the 24 h Recall Questionnaires using the Goldberg method, which represents a robust approach to assessing the accuracy and reliability of dietary intake data. This methodological validation enhances the credibility of our dietary assessments, ensuring the validity of the information obtained from participants. Secondly, the substantial number of participants. Finally, the consideration of possible confounding variables such as gender, age, TEI, physical activity, parental education, and excreted potassium [15,58].

## 5. Conclusions

This study indicates that higher values of %BF are associated with increased 24 h urinary sodium excretion levels, independent of TEI and potassium intake, in children from the ARIA study. These findings underscore the importance of promoting nutritional education from an early age in order to reduce the consumption of high-sodium food products and encourage less salt incorporation in meals. 

Taking these results into consideration, a collective commitment between the food industry and public awareness initiatives is essential to reduce sodium intake. This collective effort can play a crucial role in promoting healthier eating habits and preventing the health issues associated with excessive sodium consumption.

## Figures and Tables

**Table 1:** Main characteristics of this study sample according overweight/obesity and body fat mass percentage.

	Total, *n* = 303 (100%)	Non-Overweight/Non-Obese (*p* < 85th)	Overweight/Obese (*p* ≥ 85th)	*p*-Value	%BF (*p* < 85th)	%BF (*p* ≥ 85th)	*p*-Value
Age (years), median (25th–75th)	8 (8–9)	8 (8–9)	8 (8–9)	0.911	8 (8–9)	8 (8–9)	0.153
Sex, *n* (%)							
Female	141 (46.50%)	110 (47.60%)	31 (43.10%)	0.498	103 (49.30%)	36 (40.00%)	0.140
Male	162 (53.50%)	121 (54.20%)	41 (56.90%)		106 (50.70%)	54 (60.00%)	
Total Energy Intake (kcal), Median (25th–75th)	2178.67 (1890.28–2510.45)	2173.15 (1864.01–2519.32)	2222.42 (1943.68–2491.09)	0.518	2175.19 (1873.54–2519.66)	2199.22 (1901.25–2507.39)	0.829
24 h Urinary Sodium Excretion (mg), Median (25th–75th)	2507.00 (1909.00–3151.00)	2392.00 (1886.00–3059.00)	2725.50 (2087.25–3680.00)	0.016 *	2300.00 (1863.00–3024.50)	2760.00 (2236.75–3622.50)	0.001 *
24 h Urinary Potassium Excretion (mg), Median (25th–75th)	1681.30 (1368.50–2111.40)	1642.20 (1290.30–2072.30)	1915.90 (1485.80–2189.60)	0.020 *	1642.20 (1290.30–2052.75)	1720.40 (1466.25–2189.60)	0.057
Ratio Na/K	2.52 (1.99–3.29)	2.51 (2.00–3.26)	2.60 (1.91–3.38)	0.928	2.46 (1.98–3.21)	2.66 (2.00–3.54)	0.111
Physical Activity, *n* (%)							
<2 times/week	131 (47.00%)	100 (47.20%)	31 (46.30%)	0.985	85 (45.00%)	44 (51.20%)	0.622
2–3 times/week	108 (38.70%)	82 (38.70%)	26 (38.80%)		76 (40.20%)	30 (34.90%)	
≥4 times/week	40 (14.30%)	30 (14.20%)	10 (14.90%)		28 (14.80%)	12 (14.00%)	
Parental education, *n* (%)							
<9 years	103 (37.30%)	71 (33.80%)	32 (48.50%)	0.054	64 (34.00%)	36 (42.90%)	0.336
10–12 years	71 (25.70%)	54 (25.70%)	17 (25.80%)		49 (26.10%)	21 (25.00%)	
≥12 years	102 (37.00%)	85 (40.50%)	12 (25.80%)		75 (39.90%)	27 (32.10%)	

Note: * denotes a statistically significant association.

**Table 2:** Analysis of the association between overweightedness/obesity and body fat mass percentage (%BF) (*p* ≥ 85th) with the median of excreted sodium.

	Excreted Sodium (>2507 mg), Crude Model, OR (95% CI)	*p*-Value	Excreted Sodium (>2507 mg), Adjusted Model, aOR (95% CI)	*p*-Value
Overweight/obese (*p* ≥ 85th)	2.04 (1.18–3.50)	0.010 *	1.56 (0.82–2.96)	0.172
%BF (*p* ≥ 85th)	2.62 (1.56–4.38)	<0.001 *	2.89 (1.58–5.30)	<0.001 *

Note: Abbreviations: aOR, adjusted odds ratio. Logistic regression was adjusted for sex, age, total energy intake, physical activity, parental education, and excreted potassium. * denotes a statistically significant association.

## Data Availability

The data that support the findings of this study will be made available by the authors upon reasonable request.
